# Deep learning classification of deep ultraviolet fluorescence images toward intra-operative margin assessment in breast cancer

**DOI:** 10.3389/fonc.2023.1179025

**Published:** 2023-06-16

**Authors:** Tyrell To, Tongtong Lu, Julie M. Jorns, Mollie Patton, Taly Gilat Schmidt, Tina Yen, Bing Yu, Dong Hye Ye

**Affiliations:** ^1^ Department of Electrical and Computer Engineering, Marquette University, Opus College of Engineering, Milwaukee, WI, United States; ^2^ Joint Department of Biomedical Engineering, Marquette University and Medical College of Wisconsin, Milwaukee, WI, United States; ^3^ Department of Pathology, Medical College of Wisconsin, Milwaukee, WI, United States; ^4^ Department of Surgery, Medical College of Wisconsin, Milwaukee, WI, United States; ^5^ Department of Computer Science, Georgia State University, Atlanta, GA, United States

**Keywords:** deep learning, breast cancer classification, fluorescence image, transfer learning, explainable AI

## Abstract

**Background:**

Breast-conserving surgery is aimed at removing all cancerous cells while minimizing the loss of healthy tissue. To ensure a balance between complete resection of cancer and preservation of healthy tissue, it is necessary to assess themargins of the removed specimen during the operation. Deep ultraviolet (DUV) fluorescence scanning microscopy provides rapid whole-surface imaging (WSI) of resected tissues with significant contrast between malignant and normal/benign tissue. Intra-operative margin assessment with DUV images would benefit from an automated breast cancer classification method.

**Methods:**

Deep learning has shown promising results in breast cancer classification, but the limited DUV image dataset presents the challenge of overfitting to train a robust network. To overcome this challenge, the DUV-WSI images are split into small patches, and features are extracted using a pre-trained convolutional neural network—afterward, a gradient-boosting tree trains on these features for patch-level classification. An ensemble learning approach merges patch-level classification results and regional importance to determine the margin status. An explainable artificial intelligence method calculates the regional importance values.

**Results:**

The proposed method’s ability to determine the DUV WSI was high with 95% accuracy. The 100% sensitivity shows that the method can detect malignant cases efficiently. The method could also accurately localize areas that contain malignant or normal/benign tissue.

**Conclusion:**

The proposed method outperforms the standard deep learning classification methods on the DUV breast surgical samples. The results suggest that it can be used to improve classification performance and identify cancerous regions more effectively.

## 1 Introduction

In the United States, approximately 1 in 8 women will develop breast cancer in their lifetime (1). More than half of women who have surgery for breast cancer undergo breast-conserving surgery (BCS) or a lumpectomy (2, 3). These treatments are designed to remove all tumor tissue while keeping as much normal/benign tissue as possible. Patients may develop cancer recurrence if the cancer cells are not entirely detected and reside on the edges of the specimen tissue (positive margin). With positive margins, patients may experience additional surgery to remove the remaining cancer cells (4).

The evaluation of the intra-operative margin is currently performed by radiographic examination and confirmed by post-operative histological analysis of hematoxylin and eosin (H&E) images. As a preferable alternative, having a microscopic imaging system for margin assessment during surgery is beneficial. Real-time whole-surface imaging (WSI) of resected tissues during BCS can be provided by deep ultraviolet fluorescence scanning microscopy (DUV-FSM) (4, 5). DUV-FSM uses surface ultraviolet excitation without destructive techniques and unnecessary optical sectioning techniques to create high-quality images of specimens. These DUV images help to detect cancer cells at the edge of the surgical specimen as they show excellent color and texture contrast between cancer and normal/benign breast tissue. In previous intra-operative margin assessment research (5), texture analysis (TA) methods were used to detect tumors in ex vivo breast surgical tissues with the DUV dataset, demonstrating excellent sensitivity and specificity. However, limitations exist due to potential bias, errors during dataset construction, and reduced diagnostic accuracy for less common tissue types. Deep learning models offer an alternative solution for automated breast cancer classification of DUV images, as they can automatically learn complex patterns and hierarchical features directly from the data. This approach enables improved generalization and adaptability across diverse tissue types, overcoming the limitations of TA methods and potentially leading to more accurate and robust classification results.

In recent years, the medical domain has explored deep-learning methods for disease detection. Image classification algorithms can aid in detecting and localizing disease regions of interest (ROI). Breast cancer classification and margin assessment are major research areas that have shown promising results (6, 7). For instance (8), classified breast cancer by combining a convolutional graph network with a convolutional neural network (CNN). In their research, a modified 8-layer CNN and a two-layer graph convolutional network were fused to obtain higher classification accuracy (9). classified breast cancer with a patch-level CNN. Their study extracted spatial information of individual patches with integrated CNN and filter algorithms (10). segmented tumor and non-tumor areas with a patch-level CNN for tumor localization, which allowed deeper tissue analysis (11). adopted a patch-level CNN to classify breast cancer subtypes (12). used a patch screening method with a CNN and clustering methods. The use of patches helped differentiate the tumor types from one another (13). used a transfer learning approach to determine breast cancer grades. This study helped determine the severity of specific tumor grades with high accuracy (14). investigated the best features with transfer learning and determined the best features for predicting specific tumor grades. To deal with data imbalance (15), incorporated a transfer learning method in their study to improve classification results by using a VGG-19 model pre-trained on ImageNet weights.

The researchers mentioned above trained and tested the network on a publicly available H&E image dataset. At the moment, there is difficulty in obtaining a large training set of DUV images due to its relative novelty and practical application on a small group of subjects. Well-trained models may generalize poorly on a limited DUV dataset. To prevent the problem of overfitting, the DUV WSI images are divided into small, non-overlapping patches. In this way, the network will be able to learn better generalizations. This patch methodology boosts the training data at the patch level and helps localize cancer regions in DUV WSI for margin evaluation. A transfer learning approach is utilized to extract convolutional features from DUV patches with a pre-trainedconvolutional neural network and input them into decision-tree-based classifiers for DUV patch-level classification. As an ensemble approach, the patch-level classification results are merged with regional importance map calculations using Gradient-weighted Class Activation Mapping++ (Grad-CAM++) (16) into a single binary decision for the WSI level. The performance accuracy of the proposed approach is tested by classifying 60 images at the WSI level.

## 2 Method

**Figure 1:**
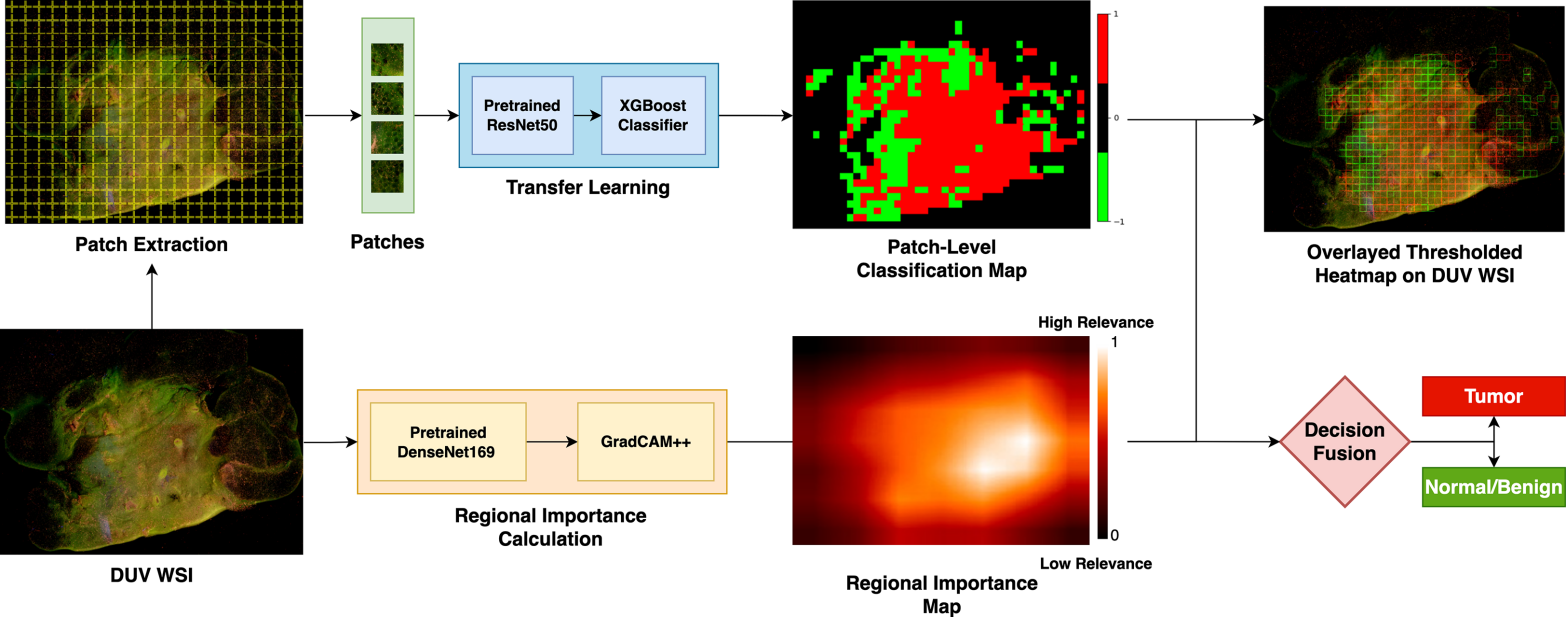
Overview of the proposed method: The WSI are divided into patches to boost the dataset and localize cancer detection. With a pre-trained ResNet50 model, the convolutional features are extracted for each patch and used to train an XGBoost classifier for patch-level classification. Grad-CAM++ on a pre-trained DenseNet169 model calculates the regional importance map for the DUV WSI. The patch-level classification results are merged with the regional importance map in a decision fusion for the WSI-level prediction.

**Figure 1** describes the proposed breast cancer classification method for DUV image margin assessment. First, DUV WSIs are divided into patches to enhance the dataset and localize cancerous regions. A pre-trained ResNet50 model (17) extracts convolutional features for each patch, which are then used to train an XGBoost classifier (18) for patch-level classification. The regional importance map for the DUV WSI is calculated using Grad-CAM++ (16) on a pre-trained DenseNet169 model (19). Finally, the patch-level classification results merge with the regional importance map in a decision fusion for the WSI-level prediction.

### 2.1 Patch extraction

A DUV WSI is divided into multiple DUV patches. The $x_{i}$ is denoted as a DUV WSI for a sample i . A 2D grid system is designed to be based on each sample’s field of view (FOV) ${\text{Ω}}_{i}$ with non-overlapping patches ${\text{Ω}}_{i}^{j}$ ,


(1) $${\text{Ω}}_{i}={\cup^{}}_{j=1}^{N}{\text{Ω}}_{i}^{j},\text{and }{\text{Ω}}_{i}^{k}\cap^{}{\text{Ω}}_{i}^{l}=\emptyset \text{ for }\forall k,l,$$

where N represents the total amount of patches in $x_{i}$ , k and l represent any patch indices.

Since each DUV WSI has different dimensions, the images are resized to the closest dimensions divisible into non-overlapping patches ${\text{Ω}}_{i}^{j}$ with a constraint size of 400×400 pixels.

The minimal alterations to the dimensions should not affect the quality of the morphological characteristics, such as cell density and infiltration, because the DUV WSI images are very large. Afterward, a 2D grid system is implemented based on each sample’s FOV ${\text{Ω}}_{i}$ with non-overlapping cells ${\text{Ω}}_{i}^{j}$ .

The patch images are extracted from each non-overlapping patch ${\text{Ω}}_{i}^{j}$ of a DUV WSI. To determine the validity of a patch, it is converted to grayscale, and its pixels are analyzed. If most of the pixels in the patch ${\text{Ω}}_{i}^{j}$ are at least 80% foreground, then it is counted as a valid patch $p_{i}^{j}$ . A pixel is a foreground if its grayscale value is greater than 5. It is important to remove the dark background to help with the localization and detection of breast cancer. This process is shown in Algorithm 1 *ExtractPatches*, and Algorithm 2 *GetPercentage*.

Algorithm 1 *ExtractPatches* is used to extract foreground patches for each DUV WSI

$x_{i}\leftarrow$
 DUV WSI sample 

i


${\text{Ω}}_{i}\leftarrow$
 2D grid system on each sample’s FOV

${\text{Ω}}_{i}^{j}\leftarrow$
 Non-overlapping patch with 
400×400
 pixels

$p_{i}^{j}\;\leftarrow$
 valid patch image

M ←
 number of DUV WSI samples
**for** 
i<M
 **do**
 Load in sample 
$x_{i}$
 **for** 
${\text{Ω}}_{i}^{j}$
 in 
${\text{Ω}}_{i}$
 **do**
  **if** GetPercentage(
${\text{Ω}}_{i}^{j}$
) 
≥ 0.2
 **then**
   Save 
${\text{Ω}}_{i}^{j}$
 as valid patch 
$p_{i}^{j}$
 for 
${\text{Ω}}_{i}$

**  end if 
 end for
end for**

Algorithm 2 *GetPercentage* is used to determine the foreground percentage of a patch 
BackgroundThreshold 
←5

ImageSize 
← 400×400
 pixels
PixelCounter 
←0

**for** 
x<400
 **do**
 **for** 
y<400
 **do**
  **if** Image[
x
][
y
] 
≥
 BackgroundThreshold **then**
   PixelCounter 
←
 PixelCounter 
+1

  **end if
 end for
end for**
 **return** PixelCounter 
/
 ImageSize

### 2.2 Patch classification with transfer learning

**Figure 2:**
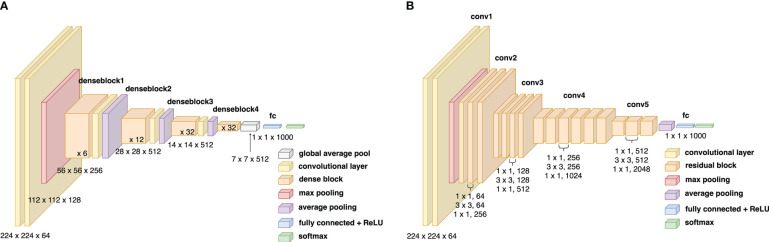
**(A)** Block diagram of the ResNet50 architecture, **(B)** Block diagram of the DenseNet169 architecture.

Convolutional neural networks are widely used for image classification problems and have the potential to diagnose diseases. In architectures like ResNet, the ‘vanishing gradient’ problem exists. The architecture of a standard ResNet50 is visualized in **Figure 2**. This happens as the increased number of layers can cause information loss of certain/specific information, preventing the network from training well. Information loss is a critical problem to overcome, especially with training with the limited DUV dataset. Even though the gradient of ResNets can transfer directly to the identity function from the previous layers to the forward layers, the summation of the identity function and output H can nullify the information flow in the network. A robust model architecture is needed to prevent overfitting for a small BCS dataset.

For this issue, a transfer learning approach determines whether each patch is malignant or normal/benign for all N DUV patches $p_{i}^{j},j=\left\{1,\ldots ,N\right\}$ . In this approach, the features are extracted from the final layer of a pre-trained ResNet50 network (17) on the ImageNet dataset and fed into an XGBoost classifier (18). The XGboost classifier predicts the binary output $y_{i}^{j}\in \left\{-1,+1\right\}$ as malignant or normal/benign for each patch $p_{i}^{j}$ . This determines the tumor ROI in the DUV WSI based on $y_{i}^{j}$ with the relative patch locations (Refer to **Figure 1**).

### 2.3 Regional importance calculation with Grad-CAM++

Grad-CAM++ (20) is an explainable artificial intelligence approach that generates visual explanations behind a model’s decisions. When applied to deep neural networks, it can visualize gradients with pixel-wise weighted feature maps. This technique explains the output layer decisions while considering the spatial information and high-level semantics from the previous convolutional layers. Since the details in the patches and the DUV images are very complex, it is crucial to implement Grad-CAM++ on a network that can retain as much information as it can. A proposed network called DenseNet (Densely Connected Convolutional Network) (19) is a modified version of ResNet where each layer is connected directly with every other layer.

As shown in this equation, the lth layer receives the feature-maps of all preceding layers, $x_{0},\ldots ,x_{\left(l-1\right)}$ , as input,


(2) $$x_{l}=H_{l}\left(\left[x_{0},\ldots ,x_{\left(l-1\right)}\right]\right),$$

where $\left[x_{0},\ldots ,x_{\left(l-1\right)}\right]$ refers to the concatenation of the feature maps produced in layers 0 to l−1 .

The novelty of DenseNet is that it removes repeated feature maps after the concatenation of the feature maps, allowing for fewer parameters. By concatenating these feature maps from different feature maps, it increases the variation in the inputs of the following layers. The bottleneck and compression layers of the DenseNet architecture are effective against overfitting due to fewer parameters needed. The bottleneck layers help reduce the number of inputs from previous k -output feature maps, which improves computational efficiency. For the compression layers, they improve model compactness by reducing the feature maps that a dense block generates. The feature maps will overlap at the last output layer, highlighting the most relevant features, which is helpful for Grad-CAM++.

The following equations describe Grad-CAM++’s process of extracting weight maps from feature maps. The final classification score is defined as $Y^{c}$ for class c as a linear combination of global average pooled convolutional feature maps $A^{k}$ in the last layer over the FOV ${\text{Ω}}_{i}$ :


(3) $$Y^{c}=\sum_{k}w_{k}^{c}.\sum_{l\in {\text{Ω}}_{i}}A_{l}^{k}$$

The gradient of the classification score $Y^{c}$ for class c before the softmax layer with respect to the final convolutional layer feature map activation $A^{k}$ is defined as $\frac{\partial Y^{c}}{\partial A^{k}}$ . The weights $w_{k}^{c}$ for a particular feature map $A^{k}$ are defined by its global average pooled gradients:


(4) $$w_{k}^{c}=\frac{1}{Z}\sum_{l\in {\text{Ω}}_{i}}\frac{\partial Y^{c}}{\partial A_{l}^{k}},$$

where Z represents the activation map’s number of pixels.

With Grad-CAM implementations, visualizations are limited if there are multiple instances of a class in the input image $x_{i}$ , as different spatial footprints of classes can cause different feature maps. The feature maps with small footprints will not be seen in the final saliency map.

To fix this issue, a weighted average of the pixel-wise gradients can be taken instead, where Equation 3 is restructured as:


(5) $$w_{k}^{c}=\sum_{l\in {\text{Ω}}_{i}}\alpha_{l}^{kc}.ReLU\left(\frac{\partial Y^{c}}{\partial A_{l}^{k}}\right),$$

where ReLU is the rectified linear unit activation function and $\alpha_{l}^{kc}$ correspond to the pixel-wise gradients for class c and convolutional feature map $A^{k}$ .

The gradient weights $\alpha_{l}^{kc}$ can be derived for a particular class c and activation map $A^{k}$ . Combining Equation 2 and Equation 3:


(6) $$Y^{c}=\sum_{k}\{\sum_{l\in {\text{Ω}}_{i}}\alpha_{l}^{kc}.ReLU\left(\frac{\partial Y^{c}}{\partial A_{l}^{k}}\right)\}\left[\sum_{l\in {\text{Ω}}_{i}}A_{l}^{k}\right]$$

Without a threshold need, the ReLU can be removed from the derivation since there is no loss of generality. Hence:


(7) $$\frac{\partial^{2}Y^{c}}{\partial {\left(A_{l}^{k}\right)}^{2}}=2.\alpha_{l}^{kc}.\frac{\partial^{2}Y^{c}}{\partial {\left(A_{l}^{k}\right)}^{2}}+\sum_{l\in {\text{Ω}}_{i}}A_{l}^{k}\left\{\alpha_{l}^{kc}.\frac{\partial^{3}Y^{c}}{\partial {\left(A_{l}^{k}\right)}^{3}}\right\}$$

To isolate $\alpha_{l}^{kc}$ , Equation 6 is rearranged:


(8) $$\alpha_{l}^{kc}=\frac{\frac{\partial^{2}Y^{c}}{{\left(\partial A_{l}^{k}\right)}^{2}}}{2\frac{\partial^{2}Y^{c}}{{\left(\partial A_{l}^{k}\right)}^{2}}+\sum_{l\in \Omega_{i}}A_{l}^{k}\frac{\partial^{3}Y^{c}}{{\left(\partial A_{l}^{k}\right)}^{3}}}$$

The Grad-CAM++ weights are calculated by substituting Equation 7 in Equation 4:


(9) $$w_{k}^{c}=\sum_{l\in {\text{Ω}}_{i}}[\frac{\frac{\partial^{2}Y^{c}}{{\left(\partial A_{l}^{k}\right)}^{2}}}{2\frac{\partial^{2}Y^{c}}{{\left(\partial A_{l}^{k}\right)}^{2}}+\sum_{l\in \Omega_{i}}A_{l}^{k}\frac{\partial^{3}Y^{c}}{{\left(\partial A_{l}^{k}\right)}^{3}}}].relu\left(\frac{\partial Y^{c}}{\partial A_{l}^{k}}\right)$$

The regional importance map $R^{i}$ for a DUV WSI $x_{i}$ is computed using a linear combination of forward activation maps.


(10) $$R^{i}=ReLU\left(\sum_{k}w_{k}^{c}.A_{l}^{k}\right),$$

This highlights the most significant features in the final classification with a positive correlation with pixel intensity and classification score with the application of the ReLU function to a linear combination of activation maps. In this study, the GradCAM++ implementation is applied on a pre-trained DenseNet169 model with ImageNet weights and extracts the feature map as the regional importance map at the Norm5 layer. The architecture of the DenseNet169 model can be visualized in **Figure 2**.

### 2.4 WSI classification with decision fusion

Given the patch-level classification labels $y_{i}^{j}\in \left\{+1,-1\right\}$ for all patches $j=\left\{1,\ldots ,M_{i}\right\}$ and regional importance map $R^{i}$ , a decision fusion method is applied to determine the WSI-level classification label $y_{i}\in \left\{+1,-1\right\}$ .

First, the regional importance $r_{i}^{j}$ is computed for each patch $p_{i}^{j}$ by taking the average value of $R^{i}$ over a patch’s FOV ${\text{Ω}}_{i}^{j}$ .


(11) $$r_{i}^{j}=\frac{1}{\left|\Omega_{i}^{j}\right|}\sum_{l\in {\text{Ω}}_{i}^{j}}R_{l}^{i},$$

where $\left|{\text{Ω}}_{i}^{j}\right|$ is the number of pixels for each patch ( 400×400 pixels).

The weight is defined as $w_{i}^{j}$ for each patch $p_{i}^{j}$ based on the thresholded regional importance value $r_{i}^{j}$ .


(12) $$w_{i}^{j}=\{\begin{array}{ll} 0\; & \text{if }r_{i}^{j}<0.25\\ r_{i}^{j}\; & \text{otherwise} \end{array}$$

This weighting scheme ignores the patches with low importance, either malignant or normal/benign, in the fused decision for the DUV WSI.

Next, the patch-level classification label $y_{i}^{j}\in \left\{+1,-1\right\}$ and weight $w_{i}^{j}$ are multiplied for each patch as $u_{i}^{j}$ .


(13) $$u_{i}^{j}=w_{i}^{j}\cdot y_{i}^{j},$$

Now, the total amount of malignant patches $H_{i}$ is calculated,


(14) $$H_{i}=\sum_{j=1}\{\begin{array}{ll} 1\; & \text{if }u_{i}^{j}>0\\ 0\; & \text{otherwise} \end{array},$$

Finally, the WSI-level classification label $y_{i}\in \left\{-1,+1\right\}$ is determined by comparing $H_{i}$ to a certain percentage q of the total foreground patches $M_{i}$ .


(15) $$y_{i}=\{\begin{array}{l} +1\;\\ \text{if }H_{i}>q\cdot M_{i},0\leq q\leq 1\\ -1\;\\ \text{otherwise} \end{array}$$

where it maps the positive (malignant) and negative (benign) value to +1 and −1 , respectively.

## 3 Results

Automated DUV WSI classification is used to determine if a sample is malignant or normal/benign tissue. The proposed method was assessed with 5-fold cross-validation. The DUV WSI data was split while managing balanced class labels and specific patient samples to stay only in training or testing data splits. There was no fine-tuning of the pre-trained ResNet50 with ImageNet weights for the Transfer Learning part. The XGBoost classifier’s hyperparameters were default settings and did not involve any tinkering. There was no hyperparameter tuning for the pre-trained DenseNet169 model with ImageNet Weights for the Regional Importance Calculation. The proposed approach was compared with a standard ResNet50 model and Patch Classification with Majority Voting. The ResNet50 (17) trained for 100 epochs on the limited DUV WSI data with a batch size of 4, a learning rate of 0.006, and a dropout of 40%. Hyperparameter tuning has been done for the ResNet50 model. The Patch Classification with Majority Voting is derived from the architecture of the proposed method’s patch classification and transfer learning portion. This approach is augmented with a patch majority voting scheme for binary classification between malignant and normal/benign for the WSIs.

### 3.1 Dataset

The breast cancer dataset consists of DUV images from 60 samples (24 normal/benign and 36 malignant). This DUV dataset was collected from the Medical College of Wisconsin (MCW) tissue bank (4) with a custom DUV-FSM system. The DUV-FSM used a deep ultraviolet (DUV) excitation at 285 nm and a low magnification objective (4X), which achieved a small spatial resolution from 2 to 3 μ m. To enhance fluorescence contrast, breast tissues are stained with propidium iodide and eosin Y. This technique produces images of the microscopic resolution, sharpness, and contrast from fresh tissue stained with multiple fluorescence dyes.

The 60 DUV images were divided into 34468 patches with a size of 400×400 pixels at 4X magnification (9444 malignant and 25024 normal/benign samples). Moreover, when the classifiers were trained, horizontal/vertical flips and 90-degree rotations were used to boost the data. The pathologists annotated and delineated tumors from the corresponding H&E images for ground-truth labels. The DUV images are registered and compared with H&E images manually. **Table 1** shows the dataset's distribution of malignant and normal/benign images in extracted patches, and DUV WSIs.

**Table 1:** Dataset Information: Extracted Patches provides the number of patches that were extracted for normal/benign and malignant.

Extracted Patches		DUV WSIs	
	Number of Patches		Number of Samples
Normal/Benign	25,024	Normal/Benign	24
Malignant	9,444	Malignant	36
Total	34,468	Total	60

DUV WSIs provides the number of samples for normal/benign and malignant.

### 3.2 Visual inspection

**Figure 3:**
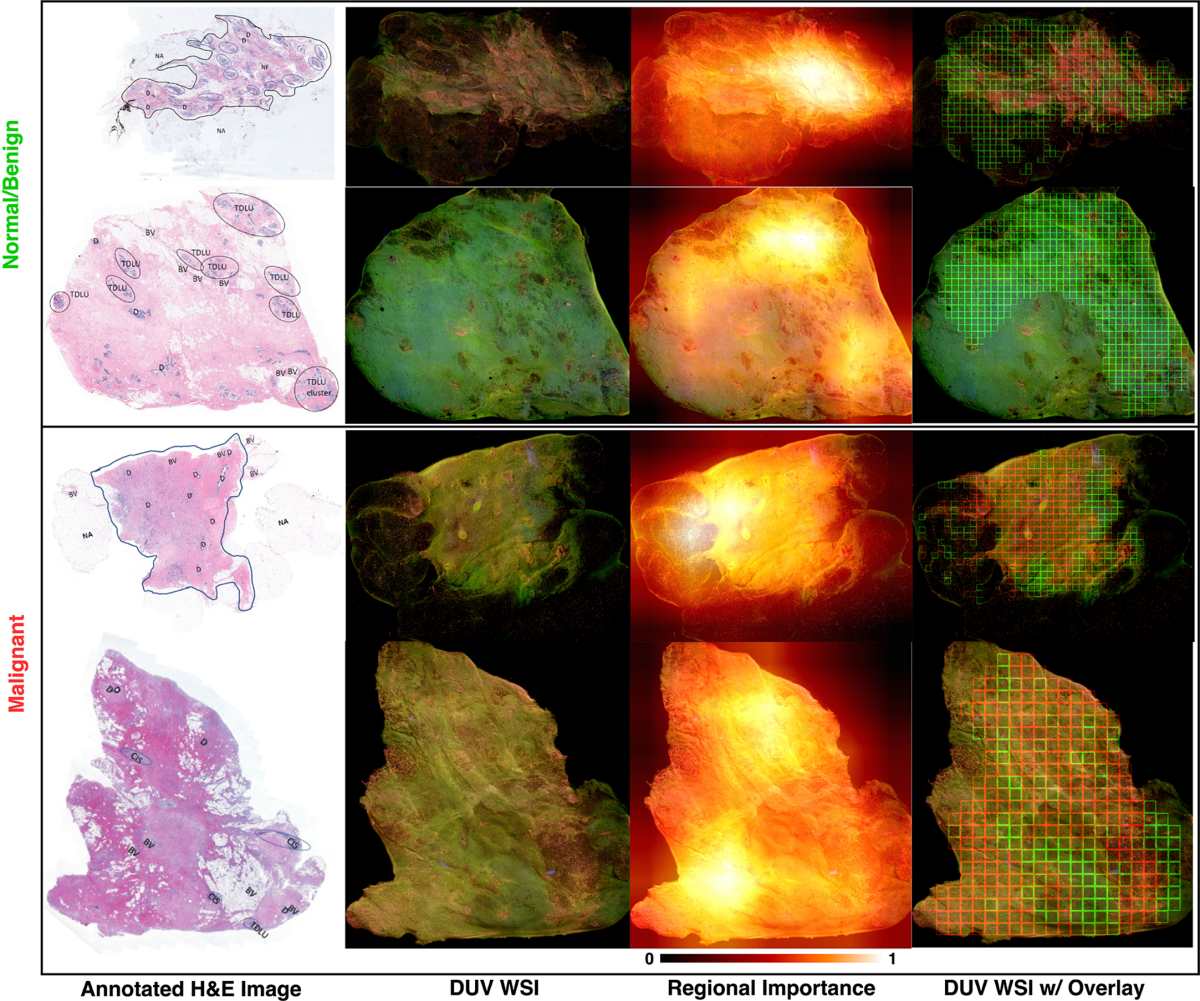
DUV WSI with corresponding H&E image and regional importance map for malignant and normal/benign samples: Regional importance areas contain high entropy, high content/semantics (3rd column). It focuses on the strong semantic areas to help in the classification. Note that the images are inputs into a pre-trained DenseNet169 with ImageNet weights, and Grad-CAM++ extracted the heatmaps where the relevancy of the tissue areas is determined from 0 to 1. Red and green bounding boxes in DUV WSIs (last column) represent malignant and normal/benign patches thresholded by regional importance, respectively. Large DUV WSIs are scaled down for visualization.

The breast cancer location and detection results on several malignant and normal/benign DUV images for qualitative evaluation are in the last column of **Figure 3**. The Grad-CAM++ influenced results are shown as the red (malignant) and green (benign) patches with high importance. The H&E images annotated by the pathologists are displayed for comparison with their DUV counterparts. In comparison, the DUV images have a higher color contrast than the H&E images when analyzing the malignant (pink/yellow) and normal/benign (light/dark green) tissues.

As seen in these DUV WSI images, the proposed method was able to accurately localize malignant and normal/benign areas with the aid of Grad-CAM++ regional importance maps. The DUV images contain overlaid regions focusing on malignant (red squares) and normal/benign adipose (green) tissue. These DUV images show that the proposed fusion method with regional importance maps can localize ROI for a WSI-level decision with confident margin prediction accuracy. The results demonstrate that even with little training data, this deep learning approach with a patching strategy can capture pathological traits like high cell density and infiltration.

### 3.3 Classification performance

The accuracy, sensitivity, specificity, and AUC score of malignant/benign binary classification on DUV-WSI images are measured for quantitative analysis. The classification performance of 5-fold cross-validation across 60 DUV-WSI images is shown in **Table 2**. The accuracy, sensitivity, and specificity were calculated with Equations 16-18, respectively:

**Table 2:** 5-fold Cross-Validation Classification Performance on 60 DUV WSI images: The proposed method (3) (Patch Classification with Regional Importance) significantly increases the classification accuracy, sensitivity, and specificity compared with the standard ResNet50 approach (1), and Patch Classification with Majority Voting (2).

	(1)	(2)	(3)
Accuracy	81.7%	93.3%	95.0%
Sensitivity	91.7%	94.4%	100%
Specificity	66.7%	91.2%	87.5%


(16) $$Accuracy=\frac{TP+TN}{TP+TN+FP+FN},$$

(17) $$Sensitivity=\frac{TP}{TP+FN},$$

(18) $$Specificity=\frac{TN}{TN+FP},$$

where TP is true positives, TN is true negatives, FP is false positives, and FN is false negatives. A confusion matrix summarizes the proposed method’s performance with the predictions against known ground truth labels in **Figure 4**.

**Figure 4:**
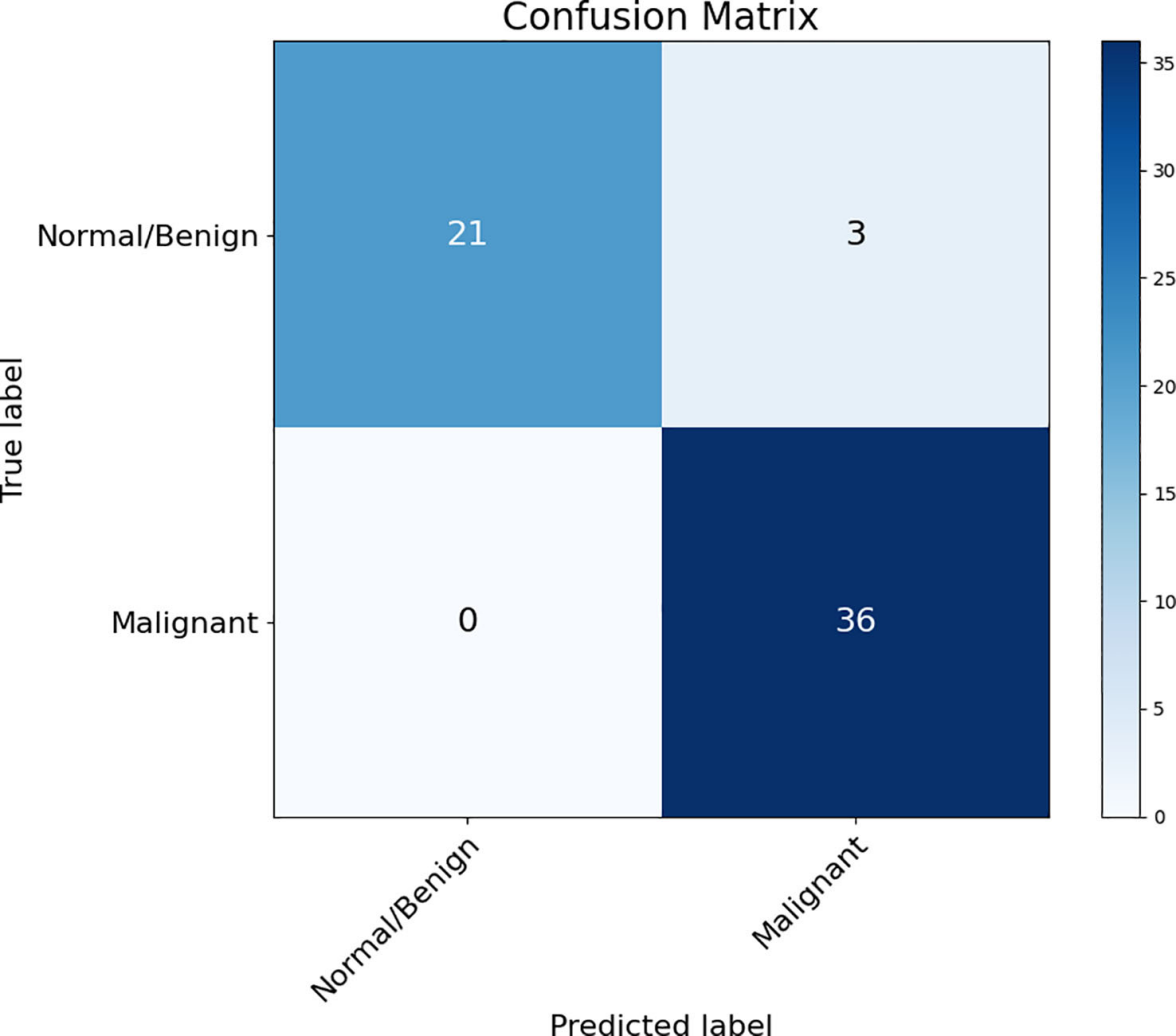
Confusion Matrix of the proposed method (Patch Classification with Regional Importance).

**Figure 5:**
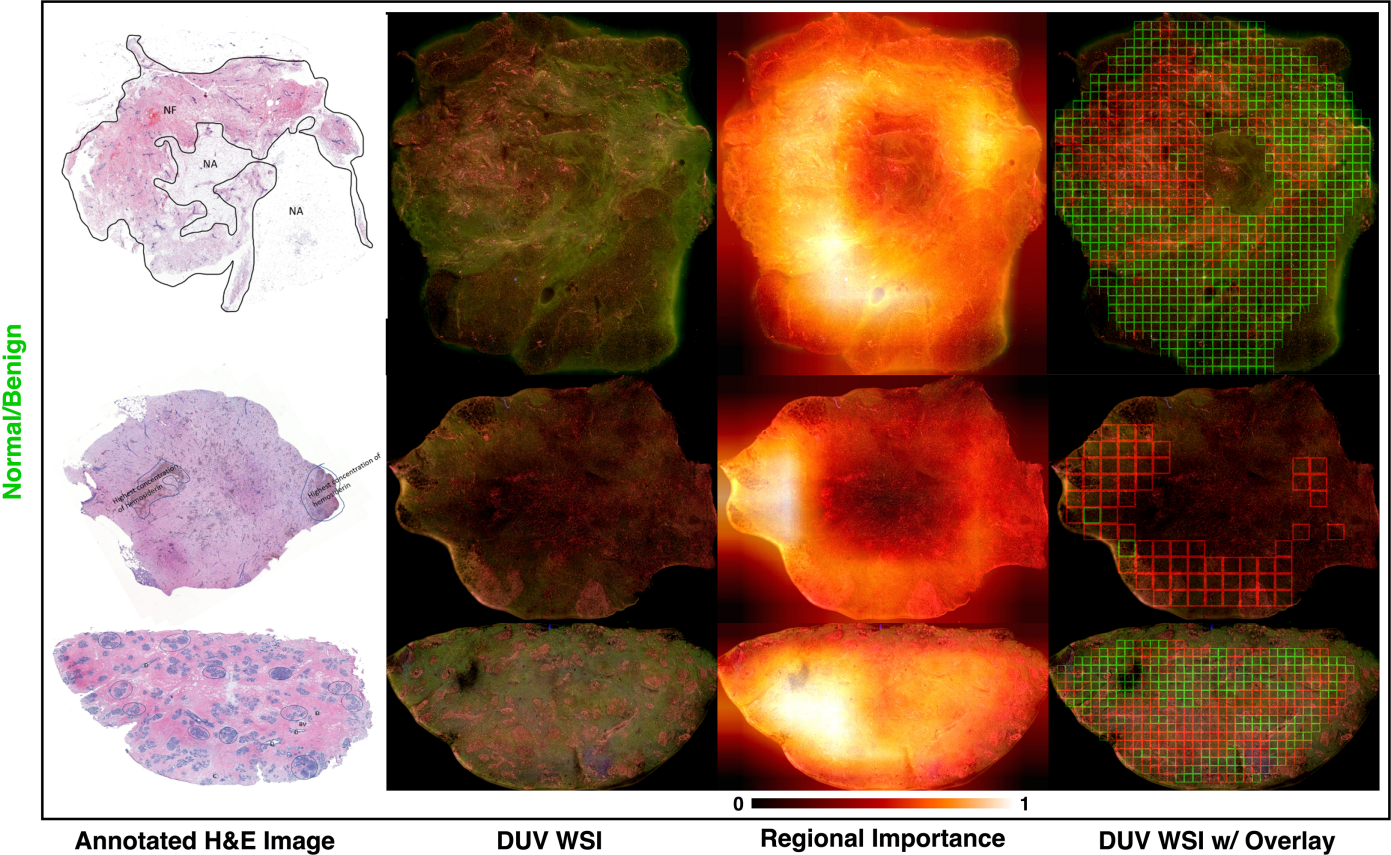
Three misclassified DUV WSI normal/benign samples from the proposed approach. H&E image and regional importance map are shown for each sample, respectively. Large DUV WSIs are scaled down for visualization.

**Figure 6:**
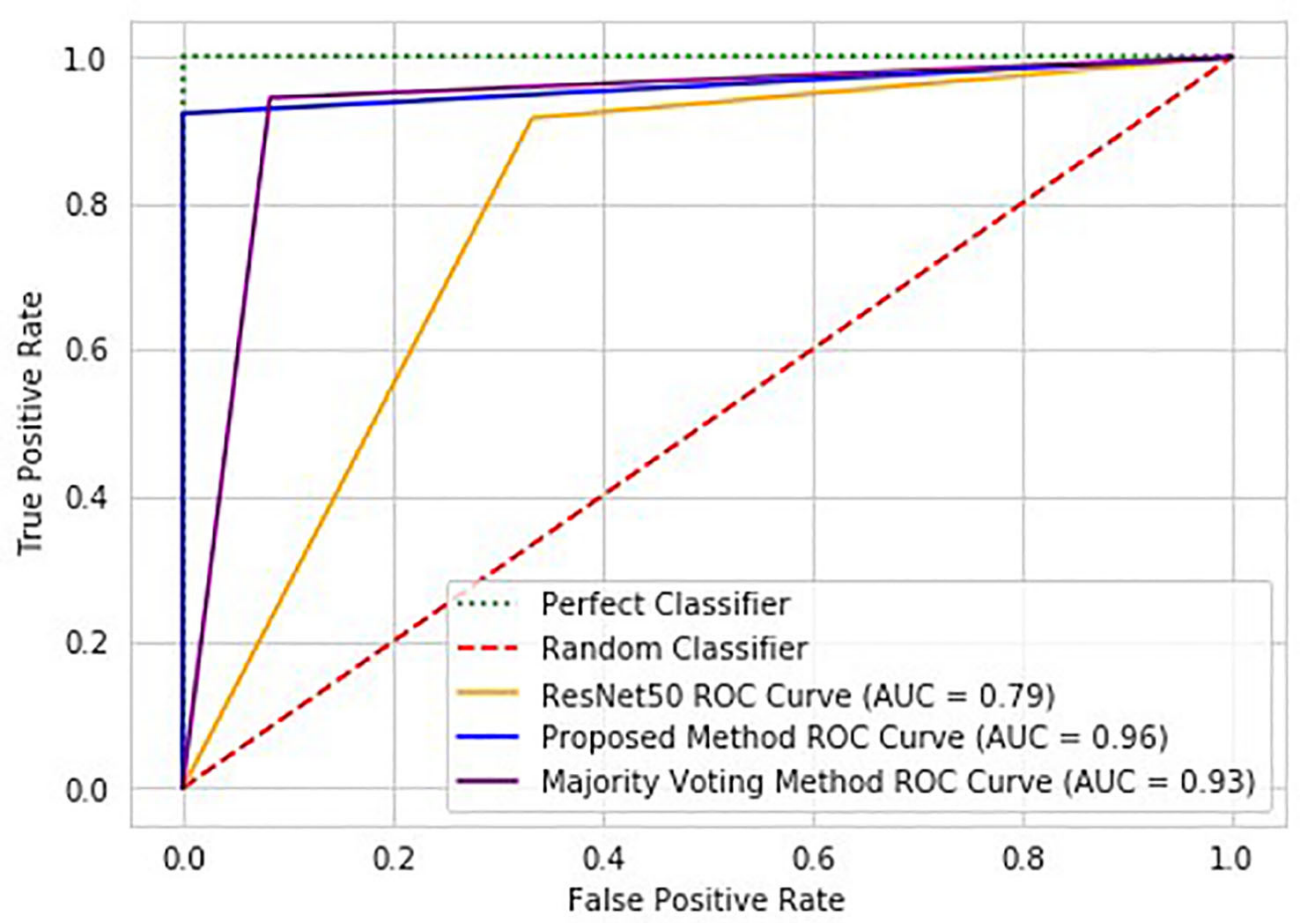
ROC graph of the proposed method (Patch Classification with Regional Importance), ResNet50 approach, and Patch Classification with Majority Voting.

Compared to standard ResNet50, the proposed method significantly improves classification performance with a 13.3% increase in accuracy. The proposed method is reliable, while the standard ResNet50 with DUV WSI has overfitting issues. The proposed method outperforms ResNet50 in sensitivity, specificity, and AUC scores by 8.3%, 20.8%, and 17.0%, respectively. **Figure 6** illustrates the proposed method’s ROC Curve, where the AUC is 17% higher than with the ResNet50 model. Compared with Patch Classification with Majority Voting, the proposed method outperforms by 1.7% and 5.6% for accuracy and sensitivity, respectively. Although the proposed method has a specificity that is 3.7% lower than another approach, it is still a good option for breast cancer classification. The proposed method achieved a perfect sensitivity rate of 100% compared to the other approach. Sensitivity is crucial in detecting malignant cases, a primary concern in breast cancer classification. The higher sensitivity rate of the proposed method makes it a valuable option despite having slightly lower specificity. These metrics demonstrate the advantage of the proposed method for intra-operative margin assessment, as it should reduce the likelihood of breast cancer margins being undetected during BCS.

### 3.4 Misclassified samples

The three normal/benign DUV WSI images that were misclassified as malignant are shown in **Figure 5**. The top sample contains a mixture of fat and fibrotic breast tissue. The dense, irregular, bumpy fibrotic tissue likely contributed to the misclassification. The middle sample has a pool of bleeding that probably resulted in misclassification. The bottom sample was considered normal/benign breast tissue with high fibro glandular density, indicating dense breast tissue. However, it is acknowledged that there may be some uncertainty in the sample classification due to other possible confounding factors. Thus, it is recommended to conduct additional analysis and confirmation to validate the classifications.

## 4 Discussion

This part discusses the results of the proposed breast cancer classification method for DUV image margin assessment, which combines patch-level classification using a transfer learning approach with regional importance maps generated by the Grad-CAM++ algorithm. The discussion involves the effectiveness of the method, its limitations, and potential future research directions to improve intra-operative margin assessment during breast-conserving surgery.

### 4.1 Methodology and results

This study used a pre-trained ResNet50 model with ImageNet weights to extract convolutional features from DUV WSI patches, which were then used to train an XGBoost classifier for patch-level classification. The regional importance maps were generated using the Grad-CAM++ algorithm on a pre-trained DenseNet169 model with ImageNet weights to localize crucial areas in the WSI image for margin assessment. The overall WSI label was determined by fusing the patch-level classification results with the regional importance map, which enhanced classification accuracy at the WSI level. The proposed method achieved an accuracy of 95% in determining DUV WSI and displayed 100% sensitivity in detecting malignant cases. Additionally, the approach could accurately localize malignant and normal/benign tissue areas, outperforming standard deep-learning classification methods on DUV breast surgical samples.

### 4.2 Comparison with existing methods

A previous study utilized a transfer learning-based approach with ResNet50 to classify H&E-stained histological breast cancer images from a different dataset (21). The method fine-tuned the pre-trained ResNet50 network with ImageNet weights to adapt it to the domain-specific breast histology image classification task using the BACH 2018 dataset, which contains 400 breast histology microscopy images with 100 samples for normal, benign, *in situ* carcinoma, and invasive carcinoma. For the experiment, 320 samples were assigned for training/validation, and 80 samples were assigned for testing with balanced classes. To address the issue of limited data and large image sizes in the BACH dataset, patches of size 512 x 512 pixels were extracted with a 50% overlap between patches, resulting in a final amount of 14,000 patches. The training/validation set was augmented by applying data augmentation through varying degrees of rotation and flipping the extracted patches. The remaining 80 samples were used as a test set to evaluate the accuracy of classification methods. The approach achieved a whole-image classification accuracy of 97.50%, and a majority voting process was employed to classify breast histology images into four tissue sub-types: normal, benign, *in situ* carcinoma, and invasive carcinoma, based on patch-wise class labels estimated using the network. This ResNet50-based method demonstrated the powerful classification capacity of transfer learning approaches for automatically classifying breast cancer histology images, especially when dealing with limited training data.

The proposed ensemble learning-based approach showcases its robustness, even with a limited dataset, offering advantages comparable to the ResNet50-based method. In contrast, the proposed method focuses on margin assessment during breast cancer surgery using the DUV WSI dataset. The method uses a pre-trained ResNet50 model with ImageNet weights to extract convolutional features from DUV WSI patches. These extracted features are then used to train and test an XGBoost classifier for patch-level classification, providing a more robust and accurate representation of the input data. Combining the pre-trained ResNet50 model and the XGBoost classifier effectively utilizes the limited dataset available while minimizing overfitting, a critical aspect when dealing with a small dataset like the one used in the study.

The proposed ensemble learning method offers several advantages compared to the ResNet50-based approach. One key advantage is incorporating regional importance maps generated using the Grad-CAM++ algorithm on a pre-trained DenseNet169 model with ImageNet weights. These regional importance maps help focus on areas with the highest level of detail and semantic information within the DUV WSI images. By capturing the spatial distribution of the most discriminative features, the algorithm highlights the most critical regions in the image responsible for the classification decision. This emphasis on semantically significant areas substantially contributes to breast cancer margin assessment, enabling the method to hone in on the most relevant image regions that could contain malignant or benign tissue.

The proposed method aims to facilitate more accurate and efficient decisions during breast cancer surgery by concentrating on the critical aspects of margin assessment. This additional step offers a more streamlined decision-making process tailored for margin assessment during breast cancer surgery. In addition to patch-level classification, the proposed approach assigns a malignant or normal/benign label to each WSI. By focusing on this binary classification, the proposed method simplifies the task for surgeons, making it easier to determine whether the margins are clear or require further resection. On the other hand, the ResNet50-based method classifies images into four tissue types: normal, benign, *in situ* carcinoma, and invasive carcinoma. While this approach provides valuable information regarding the tissue types present, it may not directly apply to margin assessment in the same way as the proposed method, as it requires additional interpretation to decide the margin status.

Although a direct comparison between the two approaches may not be fair due to their fundamental differences, the proposed method highlights the potential advantages of deep learning techniques in breast cancer margin assessment using DUV images. By utilizing transfer learning, a robust gradient-boosting algorithm, and regional importance maps, the proposed approach demonstrates the effectiveness of deep learning in accurately identifying cancerous regions in DUV WSI images. This method paves the way for further research and development in this area, potentially contributing to improved surgical outcomes and reduced breast cancer re-excision rates after BCS.

### 4.3 Limitations and challenges

The dataset used in this study was relatively small, consisting of 60 images out of 66 available images. This limitation may restrict the generalizability of this study’s findings, as the model’s performance on more extensive or diverse datasets still needs to be investigated. Training a robust network without overfitting on such a small dataset can also be challenging, requiring careful selection of training parameters and model architecture.

Another challenge encountered in this study is the presence of mixed tissue types within many tissue samples. The label assigned to each patch may only represent a portion of the area within the patch, potentially complicating the classification process. Determining the optimal patch size remains an open question; better classification results could be achieved by identifying and using an optimal patch size that maximizes the model’s discriminatory power.

This dataset mostly contained samples that were either predominantly benign/normal or malignant, with six samples exhibiting a complex mixture of benign/normal and malignant tissues being disregarded for the sake of model training. While this approach trains the model on clean and pure samples, it may limit the generalizability of the proposed method to samples with a greater complexity of benign/normal and malignant tissues. Consequently, further investigation of the model’s performance on more complex and diverse samples is essential to validate its efficacy and robustness in real-world scenarios.

### 4.4 Future research directions

Future research can validate findings using larger and diverse datasets, optimizing patch size for classification to enhance performance, and exploring alternative evaluation metrics for more comprehensive comparisons. Assessing the clinical impact of the proposed method can guide surgical decision-making, potentially reducing the need for re-excision. Integrating the proposed method into surgical navigation systems can improve precision and minimize damage to healthy tissues. Investigating the method’s performance on other medical imaging modalities can enhance its versatility and applicability to different diagnosis techniques, contributing to better patient outcomes.

## 5 Conclusion

Accurate margin assessment during breast cancer surgery is crucial for reducing breast cancer re-excision rates following breast-conserving surgery (BCS). This study addressed the challenge of locating cancerous regions in DUV whole-slide images (WSI) by proposing an automated method leveraging deep learning techniques’ power. This methodology combines patch-level classification using transfer learning with regional importance maps generated through Grad-CAM++. Focusing on highly significant regions within the images assigns a malignant or normal/benign label to each WSI with increased confidence. This approach enhances the robustness of breast cancer classification in DUV WSI images, which is essential for accurate intra-operative margin assessment. The proposed method was evaluated on a dataset of 60 authentic DUV WSI images, and an impressive classification accuracy of 95.0% was achieved. This result indicates the potential of the proposed method for real-time assessment of margin status during breast cancer surgery and potentially other types of cancer surgeries. However, it is essential to acknowledge that the study has limitations, such as the relatively small dataset size and potential generalizability challenges for more complex tissue samples. Future work could focus on expanding the dataset, optimizing patch size, and investigating alternative evaluation metrics to validate further and enhance the proposed method. The approach used in this study could impact clinical decision-making significantly. It can provide real-time guidance to surgeons during operations, ensuring they make more informed decisions about resection margins. It can also help develop personalized treatment plans. Overall, this study demonstrates the effectiveness of deep learning techniques in breast cancer margin assessment using DUV WSI images. Furthermore, this method can be integrated into surgical navigation systems to visualize cancerous regions and their boundaries in real time, improving the precision of surgical interventions and minimizing damage to healthy tissues. The potential clinical applications and the possibility for further refinement of this method offer a promising direction for future research and development in medical imaging.

## Data availability statement

The raw data supporting the conclusions of this article will be made available by the authors, without undue reservation.

## Author contributions

Conceptualization, TY, BY, and DY; data curation, TL, MP, JJ, and TS; funding acquisition, TY, BY, and DY; investigation, TT, and DY; methodology, TT, and DY; software, TT; writing—original draft, TT; writing—review and editing, BY, and DY. All authors have read and agreed to the published version of the manuscript.
